# [^18^F]Fluoromisonidazole PET in rectal cancer

**DOI:** 10.1186/s13550-017-0324-x

**Published:** 2017-09-20

**Authors:** Tanuj Puri, Tessa A. Greenhalgh, James M. Wilson, Jamie Franklin, Lia Mun Wang, Victoria Strauss, Chris Cunningham, Mike Partridge, Tim Maughan

**Affiliations:** 1 CRUK/MRC Oxford Institute of Radiation Oncology, Department of Oncology, University of Oxford, Old Road Campus Research Building, Off Roosevelt Drive, Oxford, OX3 7DQ UK; 2 Department of Radiology, Oxford University Hospitals NHS Foundation Trust, Oxford, UK; 3 Department of Cellular Pathology, John Radcliffe Hospital, Oxford University Hospitals NHS Foundation Trust, Oxford, UK; 4 Centre for Statistics in Medicine, Oxford Clinical Trial Research Unit, Nuffield Department of Orthopaedics, Rheumatology and Musculoskeletal Sciences, Botnar Research Centre, University of Oxford, Oxford, UK; 5 Department of Colorectal Surgery, Cancer Centre, Churchill Hospital, Oxford, University Hospitals NHS Foundation Trust, Oxford, UK; 6 Present address: Department of Laboratory Medicine, Changi General Hospital, 2 Simei Street 3, Singapore, Singapore

**Keywords:** Oncology, Pharmacokinetic modelling, Rectal cancer, Hypoxia, Radiotherapy, Chemoradiotherapy, PET, PET-CT, [^18^F]FMISO, Imaging, Predictive biomarker

## Abstract

**Background:**

There is an increasing interest in developing predictive biomarkers of tissue hypoxia using functional imaging for personalised radiotherapy in patients with rectal cancer that are considered for neoadjuvant chemoradiotherapy (CRT). The study explores [^18^F]fluoromisonidazole ([^18^F]FMISO) positron emission tomography (PET) scans for predicting clinical response in rectal cancer patients receiving neoadjuvant CRT.

**Methods:**

Patients with biopsy-proven rectal adenocarcinoma were imaged at 0–45 min, 2 and 4 h, at baseline and after 8–10 fractions of CRT (week 2). The first 6 patients did not receive an enema (the non-enema group) and the last 4 patients received an enema before PET-CT scan (the enema group). [^18^F]FMISO production failed on 2 occasions. Static PET images at 4 h were analysed using tumour-to-muscle (T:M) SUVmax and tumour-to-blood (T:B) SUVmax. The 0–45 min dynamic PET scans were analysed using Casciari model to report hypoxia and perfusion. Akaike information criteria (AIC) were used to compare data fittings for different pharmacokinetic models. Pathological tumour regression grade was scored using American Joint Committee on Cancer (AJCC) 7.0. Shapiro-Wilk test was used to evaluate the normality of the data.

**Results:**

Five out of eleven (5/11) patients were classed as good responders (AJCC 0/1 or good clinical response) and 6/11 as poor responders (AJCC 2/3 or poor clinical response). The median T:M SUVmax was 2.14 (IQR 0.58) at baseline and 1.30 (IQR 0.19) at week 2, and the corresponding median tumour hypoxia volume was 1.08 (IQR 1.31) cm^3^ and 0 (IQR 0.15) cm^3^, respectively. The median T:B SUVmax was 2.46 (IQR 1.50) at baseline and 1.61 (IQR 0.14) at week 2, and the corresponding median tumour hypoxia volume was 5.68 (IQR 5.86) cm^3^ and 0.76 (IQR 0.78) cm^3^, respectively. For 0–45 min tumour modelling, the median hypoxia was 0.92 (IQR 0.41) min^−1^ at baseline and 0.70 (IQR 0.10) min^−1^ at week 2. The median perfusion was 4.10 (IQR 1.71) ml g^−1^ min^−1^ at baseline and 2.48 (IQR 3.62) ml g^−1^ min^−1^ at week 2. In 9/11 patients with both PET scans, tumour perfusion decreased in non-responders and increased in responders except in one patient. None of the changes in other PET parameters showed any clear trend with clinical outcome.

**Conclusions:**

This pilot study with small number of datasets revealed significant challenges in delivery and interpretation of [^18^F]FMISO PET scans of rectal cancer. There are two principal problems namely spill-in from non-tumour tracer activity from rectal and bladder contents. Emphasis should be made on reducing spill-in effects from the bladder to improve data quality. This preliminary study has shown fundamental difficulties in the interpretation of [^18^F]FMISO PET scans for rectal cancer, limiting its clinical applicability.

**Electronic supplementary material:**

The online version of this article (10.1186/s13550-017-0324-x) contains supplementary material, which is available to authorized users.

## Background

Hypoxia in cancer cells can lead to radioresistance and influence radiotherapy (RT) response [1–3]. Strategies to improve RT outcomes by targeting hypoxia have repeatedly failed due to the inability to reliably identify tumours with severe or non-resolving hypoxia [4]. Thus, attempts to reliably identify such tumours through functional imaging or biochemical analysis are required.

Tumour hypoxia is a consequence of imbalance between oxygen consumption and supply [5] due to factors mostly related to perfusion, diffusion or anaemia. Briefly, perfusion-related acute (also known as cyclic [6]) hypoxia is caused by limited oxygen delivery to the tissue due to fluctuations in blood flow as a consequence of poor tumour microvasculature. Acute hypoxia often fluctuates with cycle times that range from a few cycles per hour to many hours or days. Diffusion-related chronic hypoxia is caused by an increase in diffusion distances of oxygen from isolated blood vessels leading to inadequate oxygen supply which lasts from a few hours to many days. The spatial characteristics of cycling hypoxia commonly involve networks of microvessels as opposed to isolated blood vessels. Anaemic hypoxia is tumour-associated or therapy-induced due to reduction in oxygen transport capacity of blood which is exacerbated by the presence of low perfusion. An arterial blood partial pressure (pO_2_) less than 80 mmHg is considered hypoxemic hypoxia [7].

The Eppendorf electrode is considered the gold standard for measuring oxygen distribution in tumours [8] and has been shown to correlate with response to RT [9–13]. This method is invasive and underestimates oxygenation [14] in heterogeneous tissue due to inability to distinguish between areas of necrosis and viable tumour. Imaging-based non-invasive method to measure distance between tumour tissue and the nearest vessels exists, which can assess the whole tumour [15, 16], but measuring the continuum of diffusion distances is rather challenging [17].

Increased retention of [^18^F]fluoromisonidazole ([^18^F]FMISO) in tumour cells pre-treatment is suggestive of hypoxia [18] and has been shown to correlate with pO_2_ polarography defined tumour hypoxia in head and neck (H&N) cancer [8, 19, 20]. [^18^F]FMISO uptake spatially corresponded with exogenous and endogenous biomarkers of tissue hypoxia in animal models [21, 22]. Decrease in [^18^F]FMISO uptake was observed in positron emission tomography (PET) images with reduced hypoxia in animal models [23]. However, [^18^F]FMISO PET showed contradictory results in detecting tumour hypoxia in human soft tissue sarcomas [24–26]. Due to the limited spatial resolution of the PET scanner, the image voxels may contain a mixture of acute and chronic hypoxia and an attempt to separate the two has also been reported [27]. This is important because chronic as well as acute hypoxia may play a role in determining the treatment outcome [28].

There is an increasing interest in developing predictive biomarkers of tissue hypoxia using functional imaging for personalised RT in patients with rectal cancer that are considered for neoadjuvant chemoradiotherapy (CRT). Therefore, the aim was to explore changes in [^18^F]FMISO PET parameters in human rectal tumours before and after 8–10 fractions of CRT to predict clinical response.

## Methods

### Patients and ethics

Patients were recruited within an ethically approved prospective observational study: modulation of Radiotherapy according to HYpoxia: exploiting changes in the Tumour Microenvironment to improve outcome in rectal cancer (RHYTHM). The trial was registered at ClinicalTrials.gov, URL: https://clinicaltrials.gov/, number: NCT02157246, date of registration: 10 October 2013, date of enrolment of first participant: 20 Dec 2013. All patients provided written informed consent for the study procedures. Patients with histologically confirmed invasive adenocarcinoma of the rectum having neoadjuvant CRT (45 Gy in 25 fractions over 5 weeks plus capecitabine chemotherapy (900 mg/m^2^ twice a day)), prior to planned curative rectal resection, were recruited between October 2013 and April 2016.

### Clinical response

Pathological tumour regression grade (TRG) was scored using American Joint Committee on Cancer (AJCC 7.0) criteria that have been shown to predict prognosis and was considered the reference for comparison against PET outcome. Response is defined as good where patients had AJCC TRG score 0/1 with N0/1 disease post-CRT or good clinical response where surgery was not undertaken. Poor responders had AJCC TRG score 2/3 or N2 nodes on resection, or poor clinical response. Clinical response is based upon imaging, endoscopy and biopsy post CRT.

### PET scans

Patients were scanned with [^18^F]FMISO PET at baseline (scan 1) and after receiving 8–10 fractions of CRT within approximately 2–3 weeks of the baseline scan (scan 2). [^18^F]FMISO was provided by the Radiochemistry Laboratory, Wolfson Brain imaging Centre, University of Cambridge, and produced based on the synthesis methods developed by Oh et al. [29] and Lim and Berridge [30]. Production failed on two occasions. In one patient, scan 2 was not obtained, and in another patient, the baseline scan could not be done and so scan 2 was omitted. Patients were asked to empty their bladder before the start of the scan and positioned head-in-first. The injection arm was raised then folded during acquisition such that the tumour site was within the centre of the field of view (FOV). The PET-CT scans were acquired on a Discovery PET-CT 690 (General Electric Medical Systems, GEMS, Milwaukee, WI) with 157 mm axial FOV [31]. Scans were started 30 s before the administration of [^18^F]FMISO with a prescribed dose of 370 (range 333–397) MBq. Dynamic PET images were acquired at 0–45 min in list-mode and static images at 2 and 4 h. Each PET scan was preceded by a CT scan, which resulted in 512 × 512 × 61 voxels of size 0.98 × 0.98 × 2.53 mm at all stages of scanning (dynamic, 2 and 4 h). PET datasets were reconstructed using time-of-flight ordered subset expectation maximisation (OSEM) protocol (GE’s VPFX) in 3D mode with two iterations, 24 subsets and 6.4 mm Gaussian filter (~ 7 mm PET spatial resolution [16]) in accordance with the standard clinical protocol at our centre [32]. Dynamic PET resulted in 128 × 128 × 47 voxels of size 5.47 × 5.47 × 3.27 mm for each of the 40 frames (1 × 30 s, 12 × 5 s, 6 × 10 s, 5 × 30 s, 10 × 60 s, 6 × 300 s). Static PET at 2 h (600 s) and 4 h (600 s) resulted in 256 × 256 × 47 voxels of size 2.47 × 2.47 × 3.27 mm. PET images were corrected for radioactive decay and for attenuation using CT. Arterialised-venous blood samples were acquired at 45 min and 2 and 4 h time-points post tracer injection [33]. Patients underwent magnetic resonance (MR) scan on the same day prior to PET.

The data split into two cohorts; the first six patients did not receive enema, and the last five were planned to receive MICROLAX® micro-enema before the 4 h PET-CT scan. The former will be referred to as the non-enema group and the latter as the enema group throughout this manuscript.

### ROI analysis

The muscle regions of interest (ROI_muscle_) were drawn over the gluteus maximus muscle (Additional file 1: Figure S1) on the CT component of PET-CT on the 4 h scan with PMOD image processing software (PMOD Technologies (v3.6) Ltd., Zurich, Switzerland). The tumour regions of interest (ROI_tumour_) were manually delineated on MR scans by JF (a Radiologist with 7 years’ experience reporting rectal MR scans), JW and TG and transferred to the PET-CT using rigid registration in Eclipse radiation treatment planning software (Varian Medical Systems (version 10), Inc., Palo Alto, CA). The ROI_tumour_ were transferred to PMOD for analysis (Additional file 1: Figure S1). The tumour regions were again modified by TP in PMOD to ensure the exclusion of bladder from the ROI_tumour_. All ROIs were propagated to the earlier scans using rigid registration in PMOD.

### Static PET quantification

The 4 h static [^18^F]FMISO PET images were analysed using tumour volume, tumour-to-muscle standardised uptake value maximum (T:M SUVmax), tumour-to-blood standardised uptake value maximum (T:B SUVmax), tumour hypoxic volume based on the threshold value of T:M SUVmax > 1.3 and T:B SUVmax > 1.3 and the corresponding percentage (%) of the total tumour volume that is hypoxic based on the corresponding threshold values.

### Dynamic PET quantification

A small ROI within the femoral arteries (ROI_artery_) was manually placed on 3–5 consecutive transaxial slices (Additional file 1: Figure S1) within PMOD, and the combined counts from both arteries were used to define the [^18^F]FMISO blood curve calibrated to the plasma concentrations. An example of a 0–4 h time activity curve (TAC) from tumour, blood and muscle is shown in Additional file 1: Figure S2. The 0–45 min data was analysed using a standard 3-tissue compartmental model (TCM) with 7 parameters (*V*_*b*_ as fractional blood volume; *K*_*1*_, *k*_*3*_ and *k*_*5*_ as forward; *k*_*2*_, *k*_*4*_ and *k*_*6*_ as backward tracer transfer rates between the compartments and *K*_*i*_ describing the net influx of tracer to the inner-most cellular compartment), a 2-tissue compartmental model (TCM) with 5 parameters (including *V*_*b*_, *K*_*1*_, *k*_*2*_, *k*_*3*_, *k*_*4*_, *K*_*i*_), 1-TCM with 3 parameters (including *V*_*b*_, *K*_*1*_, *k*_*2*_) in PMOD, and the Casciari model in MATLAB (The MathWorks (R2013b), Inc., Natick, USA). The Casciari model provided 8 parameters with the most relevant parameters including blood perfusion (*F*) and hypoxia (*K*_*a*_) (see Appendix for details). For physiological relevance, the lower-limit of fitting parameters was set at 0, the upper-limit for *η* and *α* was set to unity as these were fractional values and the upper-bound of other parameters was set at 5 to obtain results in per second units.

### Statistical analysis

Akaike information criteria (AIC) (for *n* < 40) were used to compare the data fittings between different pharm﻿acokin﻿etic models [34], where the lowest AIC value provides the best model fit. The percentage changes between different measurements pre- and during CRT were assessed from static and dynamic [^18^F]FMISO PET scans. It was assumed that the calculation of % changes pre- and during CRT may reduce systematic bias by cancelling some of the systematic errors on two occasions. The Shapiro-Wilk test [35] was used to evaluate the normality of data.

## Results

### Patients

A total of 11 patients undergoing neoadjuvant chemoradiotherapy for locally advanced rectal cancer were recruited. All patients received 45 Gy in 25 fractions with concurrent capecitabine chemotherapy (1 patient had capecitabine withheld for 10 days due to low platelets). PET scans were done in 10/11 patients at baseline and 9/11 patients after 8–10 fractions of CRT. The patient characteristics are outlined in Table 1.

**Table 1:** Patient characteristics for RHYTHM study

Median (IQR) or number of patients (%)	
Group	RHYTHM B
Number	11
Age (IQR)	67 (19)
Male	9 (82)
Tumour stage (MRI)	
T2	2 (18)
T3	9 (82)
T4	-
Nodal stage (MRI)	
N0	4 (36)
N1	6 (55)
N2	1 (9)
Metastasis stage (CT)	
M0	11 (100)
Differentiation	
Well	1 (9)
Moderate	6 (55)
Poor	1 (9)
Not specified	3 (27)
Underwent TME	8 (73)
Response to CRT^a^	
Good	5 (45)
Poor	6 (55)

*IQR* interquartile range, *CRT* chemoradiotherapy, *%* percentage, *TME* total mesorectal excision, *CT* computed tomography, *MRI* Magnetic resonance imaging, *AJCC* American Joint Committee on Cancer, *TRG* ​tumour regression grade

^a^Good response = AJCC TRG 0/1 with N0/1, or good clinical response; Poor response = AJCC TRG 2/3, N2 post-CRT or poor clinical response. One patient had delayed surgery due to initial good response, clinical outcome used

### Clinical response

Eight patients underwent total mesorectal excision. Of the 3 patients who have not had surgery, 2 had a good clinical response and remain in remission on follow-up. The other declined surgery despite residual disease and subsequently had progressive disease. Five patients were classed as good responders (AJCC TRG 0/1 with N0/1 post-CRT or good clinical response) and 6 as poor responders (AJCC 2/3, N2 disease post-CRT, or poor clinical response) as outlined in Table 1.

### Statistics

The PET parameters failed the Shapiro-Wilk normality test on a number of occasions, and therefore, non-parametric median and inter quartile ranges (IQR) were reported.

### Analysis of static [^18^F]FMISO PET scans at 4 h

**Fig. 1:**
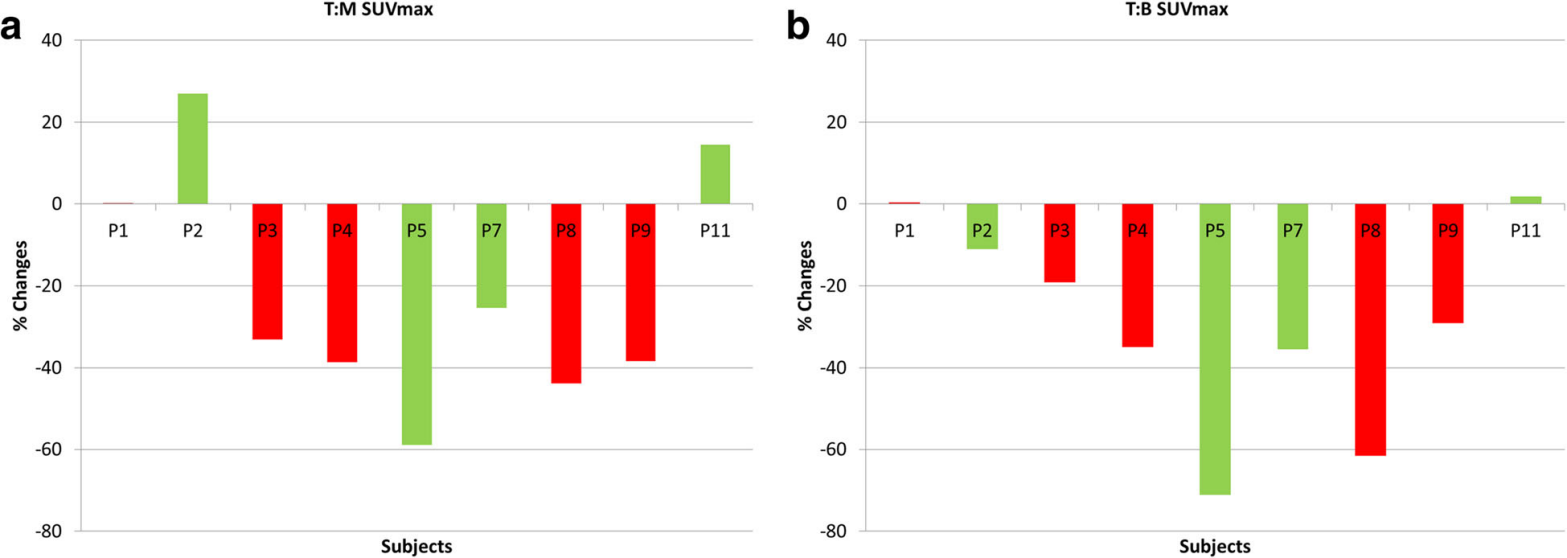
(**a**) Tumour:Muscle SUVmax and (**b**) tumour:Blood SUVmax percentage changes between baseline and after 8–10 fractions of CRT from [^18^F]FMISO PET images at 4 h. The red bars show non-responders and the green bars show responders. T:M = tumour to muscle ratio; T:B = tumour to blood ratio; SUV = standardised uptake value; max = maximum; CRT = chemoradiotherapy; [^18^F]FMISO = [^18^F]fluoromisonidazole; PET = positron emission tomography; h = hour; % = percentage

- (**a**)T:M SUVmax: Tumour hypoxic volume was defined using a threshold of T:M SUVmax ratio > 1.3 and the corresponding percentage of tumour volume that is hypoxic are outlined in Table 2. In 3 patients, T:M SUVmax (Fig. 1) rose by up to 27% (range 0.17–27.05%), whereas it fell in 6 patients with a range of 25.43 to 58.96%. The median T:M SUVmax was 2.14 (IQR 0.58) at baseline and decreased by 33% to 1.30 (IQR 0.19) by week 2. The corresponding median tumour hypoxic volume was 1.08 (IQR 1.31) cm^3^ and decreased by 95% to 0 (IQR 0.15) cm^3^ by week 2. A hypoxic tumour volume was identified in all but one patient who underwent baseline [^18^F]FMISO PET, with a range of 0–13.16%. It increased in 3 patients and reduced in 5 by week 2 CRT.
- (**b**)T:B SUVmax: Tumour hypoxic volume was defined using a threshold of T:B SUVmax ratio > 1.3 and the corresponding percentage of tumour volume that is hypoxic are outlined in Table 3. T:B SUVmax (Fig. 1) rose in 2 of 9 patients, but by less than 2%. These patients also demonstrated an increase in T:M SUVmax at week 2 CRT. For those whose values fell, the range of % change was 10.94 to 71.01%. The median T:B SUVmax was 2.46 (IQR 1.50) at baseline and decreased by 29% to 1.61 (IQR 0.14) by week 2. The corresponding median tumour hypoxic volume was 5.68 (IQR 5.86) cm^3^ and decreased by 56% to 0.76 (IQR 0.78) cm^3^ by week 2. All patients scan demonstrated a hypoxic volume at baseline (range 0.48–36.25%), which fell in 6 patients (including the 5 with a reduction in T:M SUVmax -defined hypoxic volume) at week 2.
- None of the changes in PET parameters obtained from static scans at 4 h between baseline and week 2 (from Tables 2 and 3) showed any clear trend with clinical outcome.

**Table 2:** Tumour:Muscle (T:M) SUVmax ratio and hypoxic volume at baseline and after 8–10 fractions of CRT from [^18^F]FMISO PET scans at 4 h

				Baseline						After 8–10# CRT						% Change					
Patient	pTRG	Stage	Clinical	Tumour	Muscle	T:M	Tumour	Tumour	% of	Tumour	Muscle	T:M	Tumour	Tumour	% of	Tumour	Muscle	T:M	Tumour	Tumour	% of
Number	AJCC		Response	SUV	SUV	SUV	hypoxic	ROI	tumour	SUV	SUV	SUV	hypoxic	ROI	tumour	SUV	SUV	SUV	hypoxic	ROI	tumour
				max	max	max	volume	volume	volume	max	max	max	volume	volume	volume	max	max	max	volume	volume	volume
							cm^3^	cm^3^	i.e. hypoxic				cm^3^	cm^3^	i.e. hypoxic						i.e. hypoxic
P1	1	ypT3N2	Poor	2.09	1.54	1.35	0.05	80.41	0.06	1.79	1.32	1.36	0.15	88.60	0.17	−14.35	−14.50	0.17	199.99	10.19	172.26
P2	1	ypT3N1	Good	5.19	2.30	2.26	1.93	23.20	8.32	4.97	1.73	2.87	2.93	26.85	10.93	−4.31	−24.68	27.05	51.90	15.70	31.28
P3	3	ypT3N0	Poor	3.39	1.95	1.74	0.98	179.77	0.54	2.24	1.93	1.16	0.00	86.53	0.00	−33.86	−1.00	−33.19	−100.00	−51.87	−100.00
P4	2	ypT3N0	Poor	4.16	1.74	2.39	4.03	30.66	13.16	2.70	1.84	1.47	0.20	13.35	1.47	−35.10	5.79	−38.65	−95.15	−56.46	−88.86
P5	NR	N/A	Good	4.98	1.93	2.58	3.18	62.59	5.08	1.78	1.68	1.06	0.00	55.79	0.00	−64.17	−12.69	−58.96	−100.00	−10.86	−100.00
P6	NR	N/A	Poor	3.38	2.03	1.66	0.81	18.80	4.29	-	-	-	-	-	-	-	-	-	-	-	-
P7	NR	N/A	Good	3.52	1.62	2.17	0.39	4.16	9.41	2.44	1.51	1.62	0.12	0.93	13.16	−30.54	−6.85	−25.43	−68.75	−77.65	39.80
P8	2	ypT3N2	Poor	5.31	2.35	2.27	1.17	28.43	4.13	2.49	1.95	1.27	0.00	28.61	0.00	−53.22	−16.71	−43.84	−100.00	0.60	−100.00
P9	2	ypT3N1	Poor	3.04	1.45	2.10	1.44	15.06	9.58	1.82	1.41	1.30	0.00	13.96	0.00	−39.97	−2.59	−38.37	−100.00	−7.31	−100.00
P10	0	ypT0N1	Good	-	-	-	-	-	-	-	-	-	-	-	-	-	-	-	-	-	-
P11	2	ypT3N0	Good^*^	1.94	1.74	1.11	0.00	10.98	0.00	2.40	1.88	1.27	0.00	5.23	0.00	23.87	8.16	14.52	0.00	−52.34	0.00
Median				3.46	1.83	2.14	1.08	25.82	4.68	2.40	1.73	1.30	0.00	26.85	0.00	−33.86	−6.85	−33.19	−95.15	−10.86	−88.86
IQR				1.65	0.36	0.58	1.31	38.61	7.70	0.66	0.37	0.19	0.15	42.44	1.47	25.62	13.49	38.81	100.00	52.94	131.28

Response as defined in Table 1

*[* ^*18*^ *F]FMISO* [^18^F]fluoromisonidazole, *PET* positron emission tomography, *SUV* standardised uptake value, *T:M* tumour to muscle ratio, *max* maximum, *%* percentage, *CRT* chemoradiotherapy, *IQR* interquartile range, *i.e.* that is, *ROI* region of interest, *NR* No resection, *N/A* not applicable, *cm* ^3^ cubic centimetre, *h* hour, ^*^Delayed surgery due to initial good response, clinical outcome used; *TRG* tumour regression grade, *AJCC* American Joint Committee on Cancer

**Table 3:** Tumour:Blood (T:B) SUVmax ratio and hypoxic volume at baseline and after 8–10 fractions of CRT from [^18^F]FMISO PET scans at 4 h

				Baseline						After 8–10# CRT						% Change					
Patient	pTRG	Stage	Clinical	Tumour	Blood	T:B	Tumour	Tumour	% of	Tumour	Blood	T:B	Tumour	Tumour	% of	Tumour	Blood	T:B	Tumour	Tumour	% of
Number	AJCC		Response	SUV	SUV	SUV	hypoxic	ROI	tumour	SUV	SUV	SUV	hypoxic	ROI	tumour	SUV	SUV	SUV	hypoxic	ROI	tumour
				max	max	max	volume	volume	volume	max	max	max	volume	volume	volume	max	max	max	volume	volume	volume
							cm^3^	cm^3^	i.e. hypoxic				cm^3^	cm^3^	i.e. hypoxic						i.e. hypoxic
P1	1	ypT3N2	Poor	2.09	1.32	1.58	0.39	80.41	0.48	1.79	1.13	1.58	1.27	88.60	1.43	−14.35	−14.65	0.34	225.99	10.19	195.85
P2	1	ypT3N1	Good	5.19	1.45	3.57	8.41	23.20	36.25	4.97	1.56	3.18	3.99	26.85	14.85	−4.31	7.44	−10.94	−52.62	15.70	−59.05
P3	3	ypT3N0	Poor	3.39	1.76	1.93	5.31	179.77	2.95	2.24	1.44	1.56	0.59	86.53	0.68	−33.78	−18.14	−19.11	−88.94	−51.87	−77.02
P4	2	ypT3N0	Poor	4.16	1.57	2.66	6.58	30.66	21.45	2.70	1.56	1.73	0.76	13.35	5.68	−35.10	−0.11	−35.02	−88.48	−56.46	−73.53
P5	NR	N/A	Good	4.98	1.31	3.79	14.38	62.59	22.97	1.78	1.62	1.10	0.00	55.79	0.00	−64.17	23.60	−71.01	−100.00	−10.86	−100.00
P6	NR	N/A	Poor	3.38	1.50	2.26	6.06	18.80	32.25	-	-	-	-	-	-	-	-	-	-	-	-
P7	NR	N/A	Good	3.52	1.06	3.33	1.17	4.16	28.24	2.44	1.14	2.14	0.51	0.93	55.26	−30.54	7.81	−35.58	−56.25	−77.65	95.72
P8	2	ypT3N2	Poor	5.31	1.19	4.45	10.66	28.43	37.49	2.49	1.46	1.71	1.91	28.61	6.68	−53.14	22.10	−61.62	−82.08	0.60	−82.19
P9	2	ypT3N1	Poor	3.04	1.35	2.25	2.08	15.06	13.80	1.82	1.14	1.60	1.30	13.96	9.28	−39.97	−15.35	−29.09	−37.64	−7.31	−32.73
P10	0	ypT0N1	Good	–	–	–	–	–	-	–	–	–	–	–	-	–	–	–	–	–	–
P11	2	ypT3N0	Good*	1.94	1.22	1.58	2.15	10.98	19.61	2.40	1.49	1.61	0.15	5.23	2.80	23.87	21.73	1.76	0.00	−52.34	0.00
Median				3.45	1.34	2.46	5.68	25.82	22.21	2.40	1.46	1.61	0.76	26.85	5.68	−33.78	7.44	−29.09	−56.25	−10.86	−59.05
IQR				1.65	0.24	1.50	5.86	38.61	16.00	0.67	0.42	0.14	0.78	42.44	7.85	25.62	36.37	24.64	50.83	52.94	77.02

Response is as defined in Table 1. Blood was used from blood sample activity

*[* ^*18*^ *F]FMISO* [^18^F]fluoromisonidazole, *PET* positron emission tomography, *h* hour, *SUV* standardised uptake value, *T:B* tumour to blood ratio, *max* maximum, *cm* ^*3*^cubic centimetre, *%* percentage, *CRT* chemoradiotherapy, *IQR* interquartile range, *i.e.* that is, *ROI* region of interest, *NR* No resection, *N/A* not applicable,  *TRG* tumour regression grade, *AJCC* American Joint Committee on Cancer, * Delayed surgery due to initial good response, clinical outcome used

### Analysis of 0–45 min dynamic [^18^F]FMISO PET scans

When the 0–45 min dynamic PET  data were fitted to the mathematical models, the AIC results showed that the Casciari model fitted the data better compared to 1-, 2- and 3-TCM for 17 out of 19 times in tumour (Additional file 1: Table S1) and 18 out of 19 times in muscle (Additional file 1: Table S2). An example of dynamic data fitted with different mathematical models is shown in Additional file 1: Figure S3 for tumour and Additional file 1: Figure S4 for muscle. The lower AIC values for the Casciari model corresponded to better fits, which is also visually evident from these figures.

**Fig. 2:**
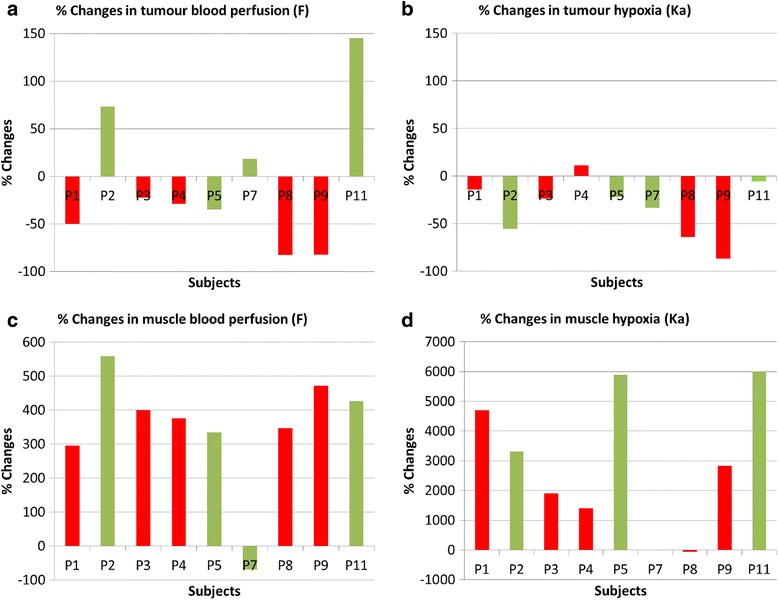
Percentage changes in tumour blood perfusion (**a**), tumour hypoxia (**b**), muscle blood perfusion (**c**) and muscle hypoxia (**d**) between baseline and after 8–10 fractions of CRT from fitting 0-45 min [^18^F]FMISO PET data to Casciari model. The red bars show non-responders and the green bars show responders. [^18^F]FMISO = [^18^F]fluoromisonidazole; PET = positron emission tomography; CRT = chemoradiotherapy; % = percentage; min = minutes

- (**a**) Tumour: The most meaningful parameters, hypoxia (*K*_*a*_) and perfusion (*F*), from the Casciari model are presented in Fig. 2 (Table 4 for details). For tumour data, the median *K*_*a*_ was 0.92 (IQR 0.41) min^-1^ at baseline and decreased by 24% to 0.70 (0.10) min^-1^ by week 2. The median *F* was 4.10 (IQR 1.71) millilitres/gram/minute ( ml g^−1^ min^−1^) at baseline and decreased by 29% to 2.48 (IQR 3.62) ml g^−1^ min^−1^ by week 2 (which is in concordance with earlier studies in solid tumours of rectal cancer (*17*)). In 9/11 patients that were scanned twice, the tumour perfusion decreased in non-responders and increased in responders except in one patient. None of the other changes in PET parameters between baseline and week 2 showed any clear trend with clinical outcome.
- (**b**) Muscle: The *K*_*a*_ and *F* from Casciari model in muscle are reported in Fig. 2 (Table 4 for details). The median *F* was 0.12 (IQR 0.04) ml g^−1^ min^−1^ at baseline and increased by 376% to 0.60 (0.12) ml g^−1^ min^−1^ by week 2. The median *K*_*a*_ was 0.02 (IQR 0.03) min^-1^ at baseline and increased by 3073% to 0.86 (0.76) min^-1^ by week 2. These values were higher than those reported by Schwartz et al. [36] perhaps due to differences in model or muscle region used. None of these changes in PET parameters between baseline and week 2 showed any clear trend with clinical outcome.

**Table 4:** Tumour and muscle imaging parameters representing blood perfusion (*F*) and hypoxia (*K*_*a*_) from fitting 45 min [^18^F]FMISO PET readings to Casciari model at baseline and after 8–10 fractions of chemoradiotherapy (CRT). Response is as defined in Table 1

		Tumour						Muscle					
Patient	Clinical	Baseline		After 8–10# CRT		% Changes		Baseline		After 8–10# CRT		% changes	
Number	Response	F	K_a_	F	K_a_	F	K_a_	F	K_a_	F	K_a_	F	K_a_
P1	Poor	0.588	1.380	0.294	1.188	−50	−14	0.144	0.018	0.570	0.864	296	4700
P2	Good	1.428	1.578	2.472	0.702	73	−56	0.102	0.042	0.672	1.434	559	3314
P3	Poor	3.198	0.912	2.484	0.696	−22	−24	0.120	0.018	0.600	0.360	400	1900
P4	Poor	4.410	0.630	3.132	0.702	−29	11	0.126	0.024	0.600	0.360	376	1400
P5	Good	6.702	0.924	4.380	0.720	−35	−22	0.138	0.006	0.600	0.360	335	5900
P6	Poor	6.702	0.924	–	–	–	–	0.600	0.444	–	–	–	–
P7	Good	3.942	0.924	4.656	0.618	18	−33	0.174	0	0.054	1.116	−69	n.d.
P8	Poor	4.368	0.870	0.750	0.312	−83	−64	0.078	4.806	0.348	2.058	346	−57
P9	Poor	4.248	0.714	0.756	0.096	−82	−87	0.084	0.036	0.480	1.056	471	2833
P10	Good	–	–	–	–	–	–	–	–	–	–	–	–
P11	Good	2.526	0.762	6.192	0.720	145	−6	0.114	0.006	0.600	0.366	426	6000
Median		4.100	0.920	2.480	0.700	−28.980	−23.680	0.120	0.020	0.600	0.860	376.190	3073.810
IQR		1.710	0.140	3.620	0.100	68.110	41.600	0.040	0.030	0.120	0.760	91.530	3225.000

*[* ^*18*^ *F]FMISO* [^18^F]fluoromisonidazole, *%* percentage, *n.d.* not defined, *IQR* interquartile range. *PET* positron emission tomography, *min* minute

### Impact of bladder activity accumulation

**Fig. 3:**
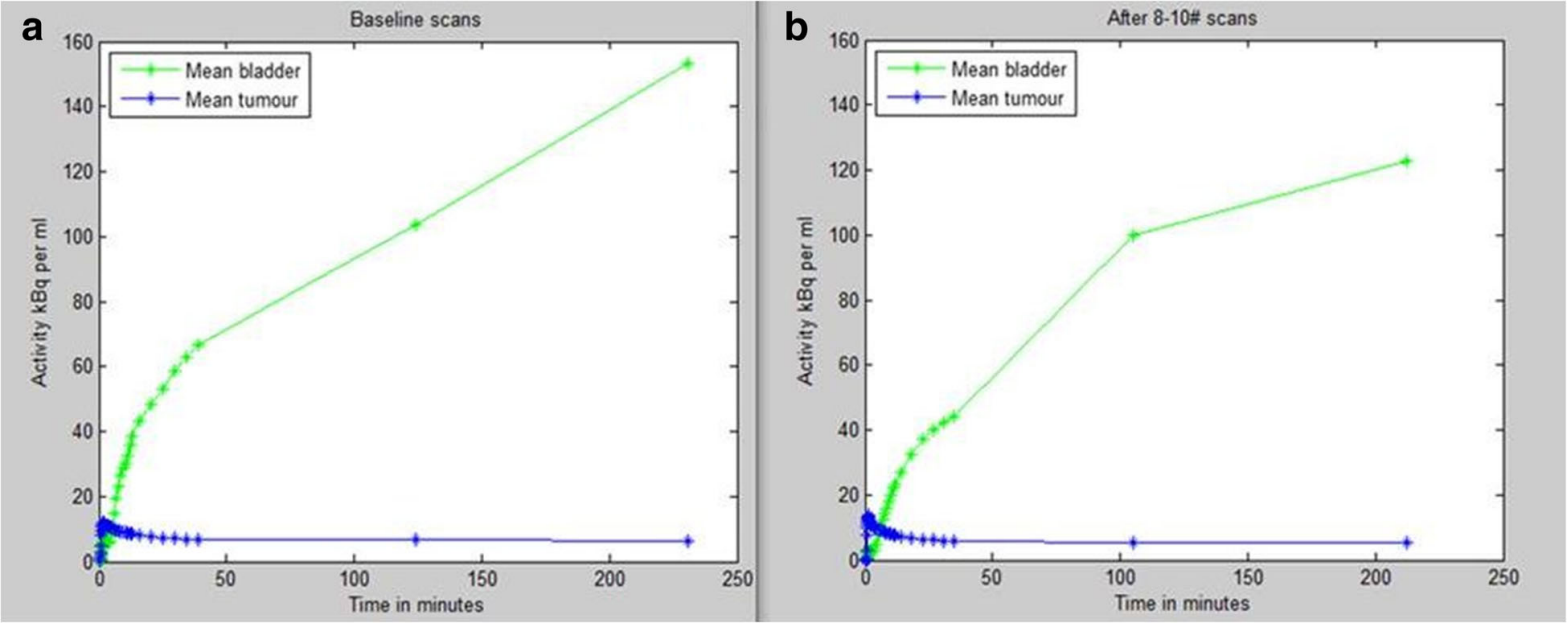
The [^18^F]FMISO PET mean time activity curve of bladder and tumour at baseline in **a** and after 8–10 fraction of radiotherapy in **b** over 0-4 h. [^18^F]FMISO = [^18^F]fluoromisonidazole; h = hour;  PET = positron emission tomography; kBq = kilo Becquerel; ml = millilitre; 1 millilitre = 1 cm^3^

It is impossible to avoid bladder emptying at some point prior to the 4 h scan. [^18^F]FMISO starts being excreted in urine by 10 min post injection leading to a high activity concentration within the bladder by the end of the study (Fig. 3 **)**.

**Fig. 4:**
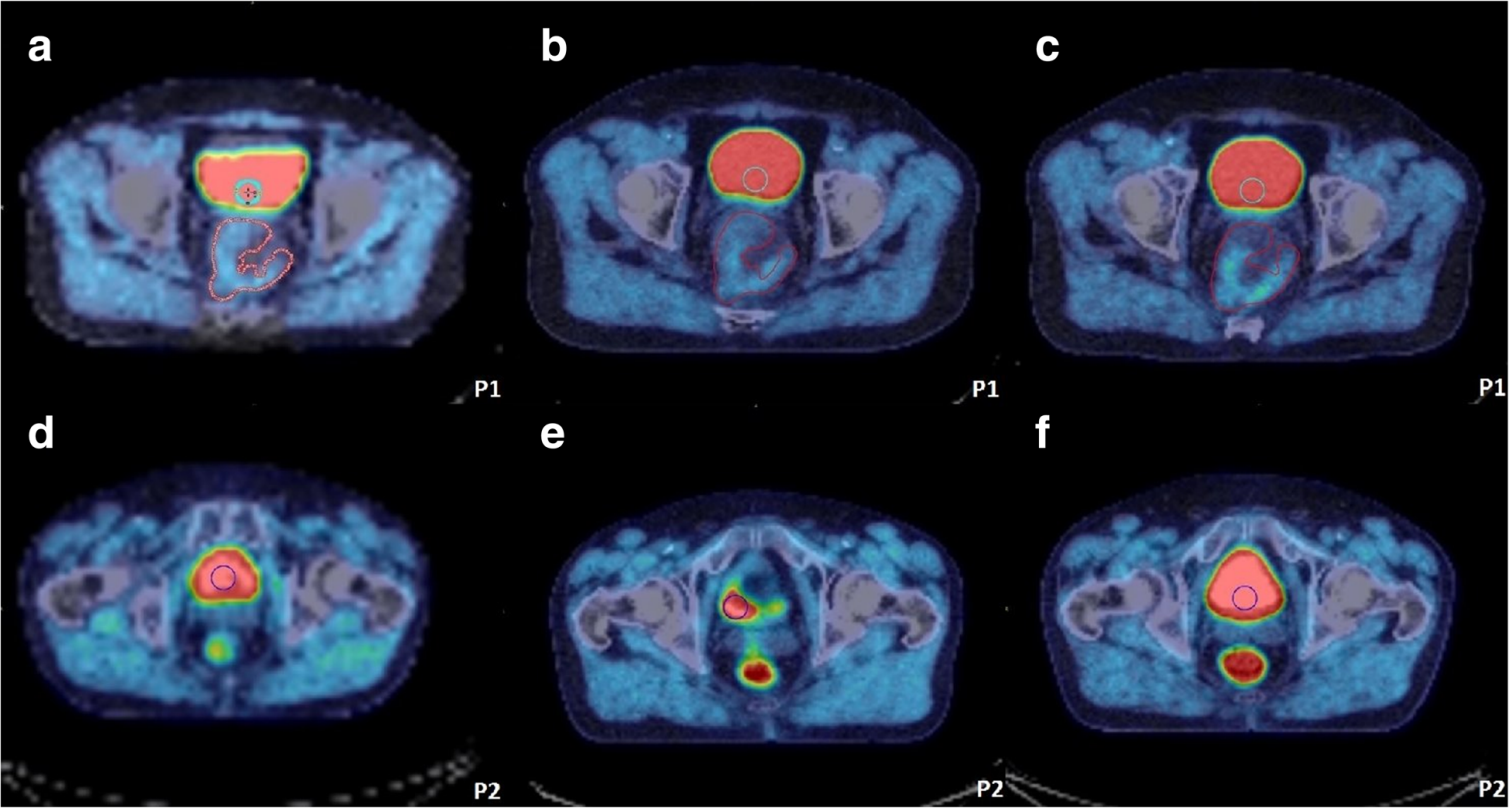
Examples from non-enema group showing PET-CT scans at 45 min (**a**, **d**), 2 h (**b**, **e**) and 4 h (**c**, **f**) for 2 subjects in whom dynamic analysis was performed. The blue circular ROI of 10 mm radius was drawn on three consecutive slices in the bladder and red ROI encompassing the whole tumour. **a**–**c** show patient 1 (P1) tumour boundary in close proximity to the bladder. **d**–**f** show that patient 2 (P2) emptied the bladder before the 2 h scan as seen from the reduced volume/activity in the bladder in **e** compared with **d** and **f**. The bladder activity reaccumulated at 4 h (**f**) compared to 2 h (**e**). The accumulation of tracer within the rectum in images **d**–**f** is two transaxial slices distal to the tumour (not visible on these slices) and is ascribed to luminal excretion of the tracer. PET = positron emission tomography; CT = computed tomography; min = minute; h = hour; ROI = region of interest; mm = millimetre

**Fig. 5:**
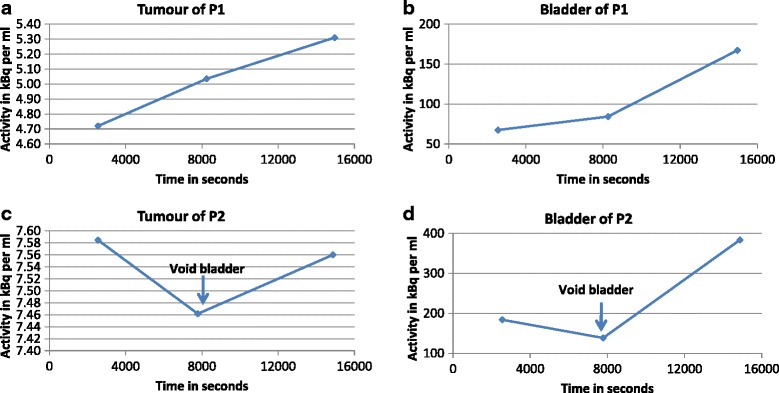
Examples from non-enema group showing TAC at 45 min, 2 and 4 h PET from tumour (**a**, **c**) and bladder (**b**, **d**). The TACs in **a** and **b** corresponds to patient 1 (P1) in Fig. 4 **a**–**c** and the TACs in **c** and **d** correspond to patient 2 (P2) in Fig. 4 **d**–**f**. The TACs show the effect of changes in bladder activity on apparent tumour activity at each time point. Although patient 2 emptied their bladder before the 2 h scan, the activity accumulated again at 4 h and consequently the activities in both bladder and tumour first dropped at 2 h (compared to 45 min) and then increased again at 4 h (compared to 2 h). This shows the effect of changes in bladder (and possibly non-tumour) activity on tumour TAC. TAC = time-activity curve; min = minutes; h = hour; PET = positron emission tomography; ml = millilitre; 1 millilitre = 1 cm^3^ kBq = kilo Becquerel

The tumour ROI can be seen in close proximity to the bladder in patient 1 as shown in Fig. 4a–c, and non-tumour accumulation of tracer posterior to bladder in patient 2 as shown in Fig. 4d–f. This suggests that the tumour TAC within the non-enema group may include contributions from spill-in from non-tumour activity in the bladder as well as rectum, in addition to hypoxic cells within the tumour. The TACs in Fig. 5a, b correspond to images in Fig. 4a–c, showing the effect of accumulated bladder activity on the tumour TAC between 45 min and 4 h, where the TAC shows a continuously increasing trend. The TAC in Fig. 5c, d corresponds to images in Fig. 4d–f showing that although the patient emptied their bladder before the 2 h scan, the activity reaccumulated at 4 h and consequently the activities in both bladder and tumour first dropped at 2 h (compared to 45 min) and then increased again at 4 h (compared to 2 h). This shows the effect of changes in bladder and non-tumour activity  in rectum on the changes in tumour TAC at each time point in the non-enema group.

### Impact of rectal activity accumulation and the effect of enema

The trial protocol was changed following the first 6 participants to mandate use of a MICROLAX® enema to be administered and the rectum emptied prior to the 4 h scan to reduce error due to excreted tracer in the rectal lumen. Four patients scanned at baseline and three scanned at week 2 underwent a microenema.

**Fig. 6:**
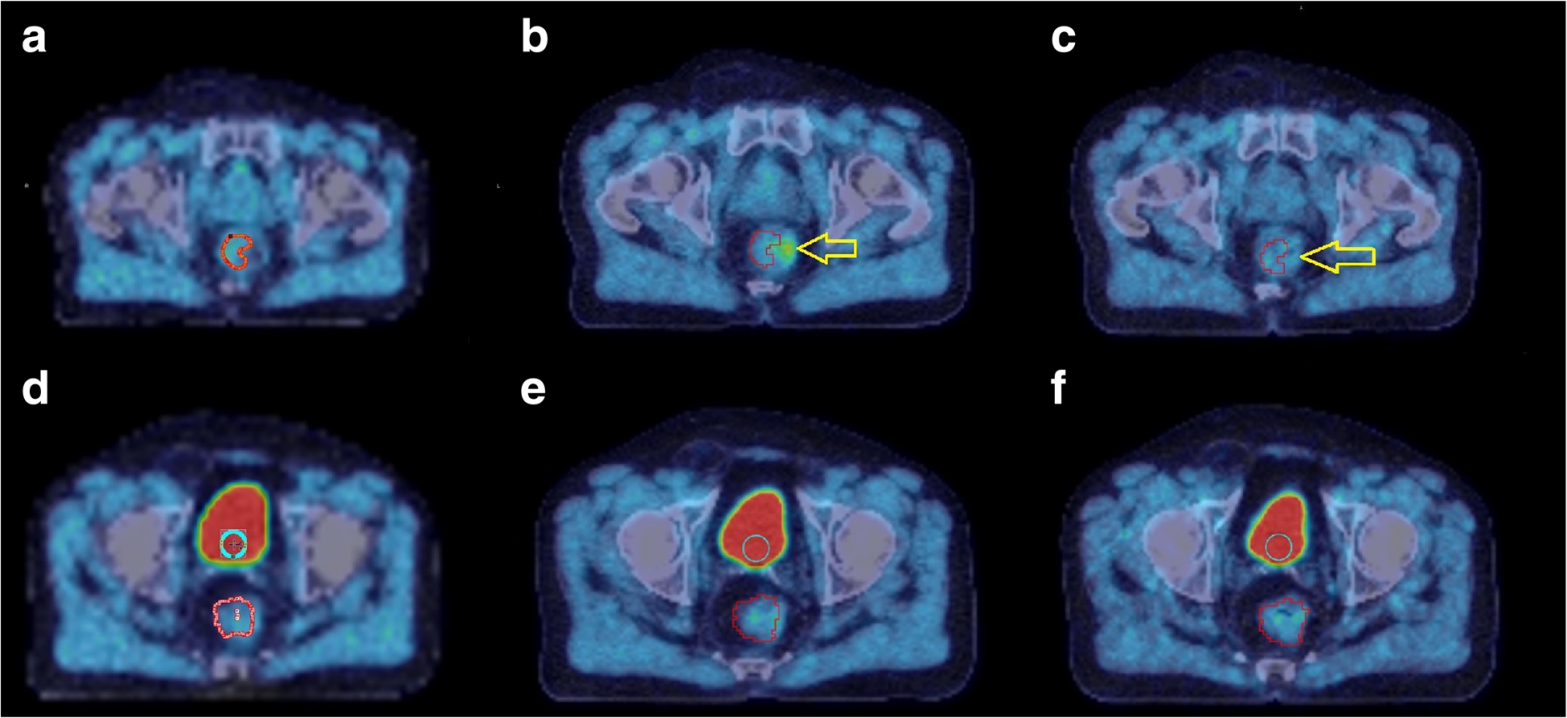
Example from enema group showing the PET-CT scans at 45 min (**a**, **d**), 2 h (**b**, **e**) and 4 h (**c**, **f**) for different transaxial slices in the same subject. The blue circular ROI of 10 mm radius was drawn on three consecutive slices in bladder, the red ROI marks the tumour and the yellow arrow shows the non-tumour activity in close proximity to the tumour ROI. **a**–**c** highlights the fact that the non-tumour [^18^F]FMISO in rectum in close proximity to the tumour is visible at 2 h, but not after the enema given before the 4 h scan. In **d**–**f**, the patient emptied their bladder before the 4 h scan as seen from the reduced volume/activity in the bladder in **f** compared to **d** (42 cm^3^ compared to 16 cm^3^ when thresholded using an absolute value of 5 kBq/cm^3^ to estimate the bladder volume) but the activity accumulated again at 4 h (in **f**). As a result, the tumour TAC in Fig. 7 shows an increase while the bladder activity was almost the same at 2 and 4 h, showing the direct effect of bladder activity on tumour [^18^F]FMISO quantification. PET = positron emission tomography; CT = computed tomography; min = minute; h = hour; ROI = region of interest; mm = millimetre; cm^3^ = cubic centimetre; TAC = time-activity curves; [^18^F]FMISO = [^18^F]fluoromisonidazole; kBq = kilo Becquerel

**Fig. 7:**
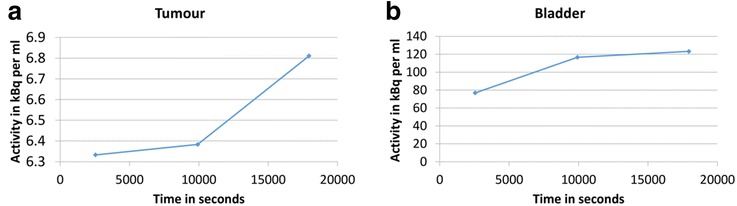
Examples from enema group showing TAC at 45 min, 2 h and 4 h PET from tumour (**a**) and bladder (**b**). The TAC in **a** and **b** correspond to patient in Fig. 6. The TACs show the effect of bladder activity on the apparent tumour activity after removal of the non-tumour activity in the rectum before the 4 h time point. The changes in tumour activity were much smaller compared to those seen in bladder as apparent from the y-axis. Since the patient emptied their bladder between 2 and 4 h, the bladder activity showed an increase of only 6% and correspondingly an increase of 6.25% in tumour activity was observed. However, it is still unclear if this increase in tumour activity is due to the spill-out from bladder or accumulation of tracer in hypoxic cells, or both. Alternatively, it is possible that the [^18^F]FMISO in hypoxic cells reached an equilibrium state by 4 h post injection, and the increase in tumour activity could be purely an effect of increased bladder activity. TAC = time-activity curve; min = minute; h = hour; PET = positron emission tomography; % = percentage; [^18^F]FMISO = [^18^F]fluoromisonidazole; kBq = kilo Becquerel; ml = millilitre; 1 millilitre = 1 cm﻿^3^

Figures 6a–c show the non-tumour accumulation of tracer in rectum adjacent to tumour at 2 h and the removal of this activity at 4 h post enema. This suggests that the 4 h PET images post-enema may be better for quantification purposes compared to those at 2 h from enema group or those at 4 h from non-enema group due to the exclusion of non-tumour activity in rectum (i.e. one less source of error). Figure 7 shows TACs derived from the images in Fig. 6 and shows the combined effect of bladder activity, non-tumour activity in rectum and hypoxic cells within tumour between 45 min and 2 h, and the effect of the enema (i.e. with non-tumour activity removed from the rectum) on 4 h PET scan and the tumour TAC. Since the patient also emptied their bladder between 2 and 4 h, the bladder activity showed an increase of only 6% and correspondingly an increase of 6.25% in tumour activity was observed. That is why it is still unclear if this increase in tumour activity in Figs. 6 and 7 post enema is due to the spill-in from bladder or accumulation of tracer in hypoxic cells or both. Alternatively, it is possible that the [^18^F]FMISO in hypoxic cells reached an equilibrium state at 4 h post injection, and the increase in tumour activity could be purely an effect of increased bladder activity.

## Discussion

Imaging with [^18^F]FMISO PET has been a valuable research tool in assessing hypoxia in various cancer types for predicting response to radiotherapy treatment in H&N and lung cancer [37–40]. In this study, we assessed the changes in [^18^F]FMISO PET parameters from static and dynamic imaging of rectal tumours before and after 8–10 fractions of CRT and reported the associated challenges.

### Analysis of static [^18^F]FMISO PET scans at 4 h

Low cellular uptake of [^18^F]FMISO in hypoxic cells and slow cellular washout from non-hypoxic tissue limits the contrast between the tissue of interest and the background at early time points post injection [41, 42]. The static PET scans at 2 and 4 h post-injection were obtained to allow tracer washout from non-hypoxic tissue [42] including muscle, which is expected to improve the characterisation between oxic and hypoxic regions inside the tumour. Timing variations occurred in acquiring 2 and 4 h PET scans with largest variation in a patient from the enema group whose 2 h PET scan was delayed by 45 min and 4 h scan delayed by 59 min.

For pixels in the tumour ROI, a tissue to blood (T:B) ratio ≥ 1.2 was considered to indicate significant hypoxia as first described by Rajendran et al. [43]. Thereafter, various authors have used T:B ratio ≥ 1.2, 1.3 or 1.4 to indicate hypoxia. There is currently no consensus on which of these values to use. Since [^18^F]FMISO concentrations in blood and muscle are nearly identical by a few minutes after tracer injection, T:M ratios were considered equivalent to T:B ratios with the benefit that no blood sampling is required. Since T:M SUVmax represented SUVmax of tumour divided by SUVmax of muscle, and since the normalisation factors in numerator and denominator cancel each other, the numerical outcome of T:M SUVmax is mathematically equivalent to the T:M ratio which is approximately equivalent to the T:B ratio (as evident from results in Fig. 1a, b). Therefore, we thought it was appropriate to use both T:M SUVmax and T:B SUVmax to define tumour hypoxia volumes.

Our tumour SUVmax values appeared higher than those obtained by Havelund et al. [44] who imaged rectal tumours with [^18^F]FAZA PET at 2 h. Tumour hypoxic volumes obtained in this study were difficult to compare with those shown by Roels et al. [42], who based rectal tumour ROI on [^18^F]FDG PET. Our T:M ratio values are however similar to those obtained in other solid tumour types such as head and neck squamous cell carcinoma [45–48] and non-small cell lung cancer [49]. Where quoted, hypoxic tumour fraction appeared higher in these tumours than in rectal cancer [48, 50].

### Analysis of 0–45 min dynamic [^18^F]FMISO PET scans

Dynamic [^18^F]FMISO PET data allows the measurement of hypoxic cells and blood perfusion simultaneously [53] which is not possible with static PET imaging at a single time point. In addition, T:B ratios from static PET scans at 4 h are likely to underestimate the hypoxia volume within a tumour, and for these reasons dynamic PET analysis was employed [16].

#### Selection of 0–45 min data

Imaging at 2 h may not be sufficient for [^18^F]FMISO to reach equilibrium within hypoxic cells [21]. The tracer cleared from the muscle at 4 h at the same time that tumour uptake starts to increase (as  shown in Additional file 1: Figure S2). This was the principal reason we first planned to analyse 0–4 h data. However, for the enema group, it was not deemed appropriate to concatenate the 0–2 h and 4 h data to obtain a combined 0–4 h tumour TAC or to combine 0–4 h data from the enema and non-enema group. This is due to the external intervention made in the enema group before the 4 h PET scan. It meant that the tumour activity at 4 h in the enema group included contributions from hypoxic cells and spill-in from bladder activity. In the non-enema group, the contributions came from hypoxic cells, bladder activity and non-tumour rectal activity. Therefore, only three 0–4 h PET datasets from the non-enema group were considered suitable for the dynamic analysis. For this reason, we decided to report the results from 0–45 min dynamic PET scans where all the datasets could be included within the analysis.

#### Arterial input function

The outcomes of mathematical modelling are sensitive to the arterial input function (AIF) [51, 52]. Since 92–96% of the [^18^F]FMISO in plasma has been shown to be intact by 90 min post-injection [53], it was thought unnecessary to correct AIF for the presence of metabolites. Continuous arterial blood sampling was not obtained in this study, and therefore, it was not possible to validate the image-derived input function from the femoral artery, which may cause bias in the estimated model parameters [54].

#### Selection of Casciari model

A few tumour and muscle TACs failed to fit using 1-, 2- and 3-TCM which could either be due to an inappropriate [^18^F]FMISO pharmacokinetic model or due to inconsistencies in the TACs. Therefore, fitting gluteus muscle data successfully to the Casciari model suggested that the tumour data needed further analysis and it was absolutely necessary to seek a pharmacokinetic model that fitted the tumour data successfully. Even though more parameters may be required to fit more complex data successfully, this does not mean that the outcome is more meaningful just because the data has been fitted by a more complex model [21]. The use of the Casciari model was based on previous recommendations [21, 55] that assumed an inverse relationship between hypoxia and perfusion within the tumour.

#### Parameter initialisation

This was a critical step in model fitting, and we used the Casciari recommended initial values. However, in some cases, different initial values were attempted to obtain better fit. It was also observed that multiple solutions existed. Nonlinear optimization may suffer from local minima and initialisation which may be minimised using stochastic sampling as suggested by Shi et al. [21].

#### Parameter fixing

The parameter fixing was undertaken to improve the precision of model parameters [21, 56]. In our case, parameter fixing either failed or gave different results and therefore was not considered any further. In the absence of true physiological values of these parameters, fixing may bias model estimates, for example, in case of rats, a large *η* value of 0.90 was obtained by Casciari [55].

#### Parameter interpretation

Baseline median *K*_*a*_ in tumour was 46 times higher than in muscle suggesting that tumour is likely to contain hypoxia cells whereas resting muscle is well oxygenated. On the other hand, baseline median *F* in tumour was 34 times higher than in muscle suggesting difference in tumour and muscular vasculature.

- (**a**) Tumour showed a median 29% and 24% decrease in *F* and *K*_*a*_ respectively after 2 weeks of CRT. However, a reduction in *K*_*a*_ post treatment can be indicative of reduced tracer delivery  due to damaged capillary bed rather than a reduction in tumour hypoxia [57]. Tumour *K*_*a*_ did not show any relationship with *F*. This suggests that the changes in median *K*_*a*_ in tumour during treatment were not due to the direct consequence of changes in perfusion and may suggest a radiotherapy-induced reoxygenation [50, 58] as it starts early during CRT and has been previously shown to correlate with outcome in H&N tumours [59, 60]. In our study, the alterations in tumour perfusion trend with response highlighted the importance of changes in vasculature-related functional parameters during radiotherapy, its important role in understanding hypoxia [61] and its relation with outcome [59, 62, 63].
- At baseline, tumour *F* showed a weak relationship with T:M SUVmax and T:B SUVmax at 4 h and none after 2 weeks of CRT, suggesting that these parameters from static PET may primarily exhibit chronic hypoxia. The *K*_*a*_ showed poor relationship with T:M SUVmax and T:B SUVmax at baseline and after 2 weeks of CRT suggesting that the semi-quantitative and quantitative method of measuring hypoxia from static and dynamic PET, respectively, are not equivalent [16].
- (**b**) At baseline, the muscle showed low *F* values and negligible *K*_*a*_ levels as expected. Despite a median increase of 376% in *F* at week 2 CRT, the median *F* in muscle was 4 times lower compared to that in tumour. The *K*_*a*_ in muscle increased by a median value of 3076% at week 2, but the median *K*_*a*_ in muscle was of the order obtained in the tumour. Acute effects of radiotherapy include reversible inflammation occurring in actively proliferating cells [64]. It is therefore likely that the muscle underwent radiation response causing inflammation in this region [64, 65]. The mechanism between inflammation and hypoxia is not well understood [66, 67], but increased perfusion due to inflammatory changes secondary to radiotherapy have been reported in rectal cancer [68]. It is also known that the [^18^F]FMISO reduction to nitro radical is reversible in the presence of oxygen. It is possible that the prolonged duration of increased blood perfusion beyond 45 min increased the accumulation of tracer in muscle during the first 2 h post tracer injection which then reversed between 2 and 4 h when we observed a large washout of tracer suggesting that the trapping mechanism is rather time dependent and fluctuating. The pattern of blood perfusion in acute hypoxia can alter on a 20 min time scale [69], which is less than our experiment time of 45 min for dynamic analysis. In our study, muscle *K*_*a*_ and *F* showed weak relationship suggesting that the muscle [^18^F]FMISO uptake was not affected by the changes in muscle perfusion [66].

### Impact of bladder activity accumulation

The accumulation of [^18^F]FMISO in bladder starts 10–15 min post-tracer injection (Fig. 3) due to variable urinary excretion and may affect tumour uptake by a phenomenon called spill-in which can affect both lesion detectability [70, 71] and quantification [72]. Additional file 1: Figure S5 shows the phenomenon of spill-in count from bladder into the tumour due to scatter and random photons. The false lines of responses are accepted as true events leading to overestimation of activity concentration in the surrounding region leading to an error during image reconstruction. Bettinardi et al. [31] assessed random/scatter characteristics over a range of 0–60 kBq/cm^3^ within the PET field of view. However, it should be noted that we observed much higher activity concentrations (undecay corrected activity of up to 150 kBq/cm^3^) within human bladder at 2 and 4 h post injection which would lead to an increase in random/scatters in a non-linear fashion. Additionally, the regions within very close proximity of the bladder gets further affected during image reconstruction by the spill-in of activity from bladder due to the limited spatial resolution of the PET scanner. The PET quantitative values within tumour may further get affected depending upon, but not limited to, the rate of urine excretion to bladder, the activity concentration within the bladder, its volume and proximity to the tumour at the time of imaging, the accuracy of attenuation correction, etc.

Catheterization of the bladder has been suggested as an invasive alternative to this problem, but it is a potential source of infection and uncomfortable for patients [73]. In addition, for radiotherapy planning purposes, a full bladder is required and so this would be unhelpful if these scans were to be used for guidance. Even though some preliminary phantom work has been done to minimise this problem by restricting the bladder counts in image reconstruction process, but it is still at an early stage to be translated to clinical data [72]. More work is required to address the PET image reconstruction [74–76] related issues before [^18^F]FMISO PET can be employed for the quantification of rectal cancer.

### Impact of rectal activity accumulation and the effect of enema

**Fig. 8:**
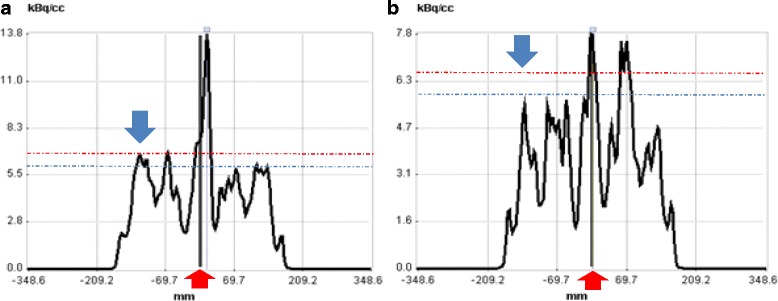
The line profiles that pass through the tumour and non-tumour activity in a patient scan at 2 h before enema as seen in Fig. 6b (in **a**) and at 4 h after enema as in Fig. 6c (in **b**). The red arrow shows the non-tumour region, and the blue arrow shows the surrounding region that encompassed the tumour. This figure shows that enema was helpful in reducing the non-tumour activity concentration from 13.8 kBq/cm^3^ (in **a**) to 7.8 kBq/cm^3^ (in B), a decrease of 44%. Consequently, the activity concentration in the surrounding region was also reduced from 7 kBq/cm^3^ (in **a**) to 6 kBq/cm^3^ (in **b**), a decrease of 14%. The red and blue dotted lines show the same numeric values on y-axis; h = hour; kBq = kilo Becquerel; CC or cm^3^ = cubic centimetre; mm = millimetre;   % = percentage

[^18^F]FMISO is excreted through the biliary tract into the gastrointestinal tract as well as through the urine. When rectal lumen contains high [^18^F]FMISO activity and it is within close proximity of the tumour, the spill-in of activity from rectal lumen into the tumour is unavoidable due to the limited resolution of the PET scanner. The use of an enema shortly prior to the 4 h scan may reduce this non-tumour luminal activity as shown in Figs. 6 and 8.

### Variability of ROI due to image co-registration

Tumours are generally adjacent to normal rectal wall and therefore difficult to outline on CT. Therefore, tumours were first outlined on MR scans and then modified to match 4 h PET uptake since there is inevitable time difference between the PET-CT and MR scans obtained on the same day. Rigid registration was used to transfer the ROI between dynamic and static scans. This should be done with caution as the internal organ motion due to air, rectal peristalsis or other reasons may not allow accurate delineation of the tumour (Additional file 1: Figure S6**)**. Since the tracer uptake in tumour changes over time, the data may benefit from careful delineation of tumour region at each time point, albeit controversial due to the time and cost involved. In the case of Additional file 1: Figure S6, it was possible to visually identify the tumour on the 4 h scans but not the 2 h scans due to the patient empting their bladder and the clearance of air in at least one patient. PET-MR may be useful in such situations.

### Other PET tracers

[^18^F]FMISO has been shown to have lower tumour uptake compared to other hypoxia tracers [77] and therefore at a disadvantage, providing lower tumour to background contrast. The PET tracers that show clearance to bladder or gut lumen are likely to have similar issues as presented within this work. A recent report [78] summarised clinical image finding for PET hypoxia tracers and suggested the use of variety of tracers in tumours of the brain, H&N, breast, lung and lymphoma. On the basis of favourable preclinical or metabolic data, the use of [^18^F]HX4 for imaging liver and [^x^Cu]Cu-ATSM for imaging bladder was also recommended even though the retention of radio-copper in animal models at early time post-injection has been shown to partially reflect processing of copper rather than a sole hypoxia marker [79]. However, limited use of most PET tracers was suggested for the assessment of hypoxia in the tumours of the renal, liver, colorectal, bladder and prostate, with the exception of [^18^F]FAZA and [^x^Cu]Cu-ATSM for colorectal tumours for visualisation purposes, though semi-quantitative methods were attempted [44, 80]. [^18^F]FMISO in colorectal was not recommended and our finding suggest the same [78]. [^18^F]FDG PET has been useful in the management of rectal cancer as a marker of glucose metabolism but not hypoxia directly and therefore is not discussed here.

## Conclusions

Since the TRG scores were obtained in 8 patients and clinical response in 3 patients who did not undergo surgery, the conclusions drawn from this study are limited by the small number of subjects. The acceptability of procedures to patients and clinical implementation is a major challenge in clinical imaging trials. Despite these challenges, some problems are solvable, for example, the spill-in from non-tumour accumulation of [^18^F]FMISO was improved using enema, while other problems may not be currently solvable, for example, the spill-in from bladder. Given the high activity regions within the pelvis, similar challenges in analysing and interpreting [^18^F]FMISO uptake may exist for other tumours, such as cervix and prostate cancer. Frusemide may be considered to aid rapid urinary excretion and dilute the residual radioactivity in the bladder. Another  solution may be to keep patients well hydrated and request bladder emptying immediately before the PET scan. However, for target delineation purposes, it is preferred that the bladder has the same size and shape as on scans acquired for radiotherapy planning i.e. ‘comfortably full’ for the registration algorithm to work well. Since CT is unable to provide a good contrast in the soft tissue within the pelvis, PET-MR may be potentially beneficial albeit controversial due to the lack of attenuation correction in the bone.

In conclusion, this pilot study with small datasets revealed significant challenges in delivery and interpretation of [^18^F]FMISO PET scanning for rectal cancer. Preliminary data does not support the hypothesis that a reduction in [^18^F]FMISO uptake is predictive of clinical response. There are two principal problems namely spill-in from non-tumour activity in the rectum and from bladder into the surrounds that encompass tumour. Careful consideration should be given to PET acquisition and reconstruction to minimise spill-in counts from the bladder. This preliminary study has shown fundamental difficulties in interpretation of [^18^F]FMISO PET for rectal cancer, limiting clinical applicability.

## Appendix

Casciari et al. [54] developed a compartmental model for [^18^F]fluoromisonidazole ([^18^F]FMISO) as following:

$$ \frac{\mathrm{d}{\mathrm{C}}_{\mathrm{c}}}{\mathrm{d}\mathrm{t}}=\frac{F}{{\mathrm{V}}_{\mathrm{t}}}\times \left({\mathrm{C}}_{\mathrm{p}}-{\mathrm{C}}_{\mathrm{c}}\right)-\left(1-\eta \right)\times {\mathrm{K}}_{\mathrm{a}}\times {\mathrm{C}}_{\mathrm{c}} $$

$$ \frac{\mathrm{d}{\mathrm{C}}_{\mathrm{bp}}}{\mathrm{d}\mathrm{t}}=\upalpha \times {\mathrm{K}}_{\mathrm{a}}\times {\mathrm{C}}_{\mathrm{c}} $$

$$ \frac{{\mathrm{C}}_{\mathrm{dp}}}{\mathrm{dt}}=\left(1-\alpha \right)\times {\mathrm{K}}_{\mathrm{a}}\times {\mathrm{C}}_{\mathrm{c}}-{\mathrm{K}}_{\mathrm{b}}\times {\mathrm{C}}_{\mathrm{dp}} $$

$$ \frac{\mathrm{d}{\mathrm{C}}_{\mathrm{d}\mathrm{pe}}}{\mathrm{d}\mathrm{t}}=\left(\frac{1-\eta }{\eta}\right)\times {\mathrm{K}}_{\mathrm{b}}\times {\mathrm{C}}_{\mathrm{c}}-\left(\frac{F}{\eta \times {\mathrm{V}}_{\mathrm{t}}}\right)\times {\mathrm{C}}_{\mathrm{d}\mathrm{pe}} $$

$$ A(t)={\beta}_1\times {C}_p+{\beta}_2\times {V}_t\times \left\{{C}_c+\left(1+\eta \right)\times \left({C}_{bp}+{C}_{dp}\right)+\eta \times {C}_{dp e}\right\} $$

The total [^18^F]FMISO activity *A*(*t*) in tissue, *F* is blood perfusion (microvascular blood flow rate measured per unit volume in units: ml g^−1^ min^−1^), *K*_*a*_ is the cellular [^18^F]FMISO reaction rate (inversely related to oxygen concentration) that is related to the fraction of cells in the region that are hypoxic (units: min^−1^), *K*_*b*_ is the rate constant for transport of diffusible products out of the cell (units: min^−1^), *V*_t_ is tissue specific volume (units: mlg^−1^), *η* is the fraction of *V*_*t*_ occupied by extracellular space (units: dimensionless), *α* is the fraction of the hydroxylamine reactive intermediate that is converted to bound products (units: dimensionless), *β*_*1*_ (units: dimensionless) and *β*_*2*_ (unit: dimensionless) were the partial volume and spill-in correction factors.

## Additional file

Additional file 1: Table S1: Tumour AIC values from fitting 0-45min dynamic [^18^F]FMISO PET readings at baseline and week 2 to four different pharmacokinetic models. **Table S2.** Muscle AIC values from fitting 0-45 min dynamic [^18^F]FMISO PET readings at baseline and week 2 to four different pharmacokinetic models. **Figure S1.** Example of the ROI from which measurements were taken. **Figure S2.** Examples of TAC in tumour, blood and muscle at baseline over 0-4 h. **Figure S3.** Example of 0-45 min tumour TACs fitted to four different pharmacokinetic models. **Figure S4.** Example of 0-45 min muscle TACs fitted to four different pharmacokinetic models. **Figure S5.** The artwork shows examples of scatter & random events originating from the activity inside the bladder as a potential cause of spill-in counts inside the tumour. **Figure S6.** An example from the enema group showing transaxial PET/CT images at 2 and 4 h. AIC=akaike information criteria; min= minute; [^18^F]FMISO=[^18^F]fluoromisonidazole; PET=positron emission tomography; CRT=chemoradiotherapy; ROI=region of interest; TAC=time activity curve; h=hour; CT=computed tomography. (PDF 445 kb)
