# Supplementary material for: [18F]Fluoromisonidazole PET in rectal cancer
Source: EJNMMI Res. 2017 Sep 20;7:78. doi: 10.1186/s13550-017-0324-x (PMC5607050; doi:10.1186/s13550-017-0324-x)
Supplement: Additional file 1: Table S1: — Tumour AIC values from fitting 0-45min dynamic [18F]FMISO PET readings at baseline and week 2 to four different pharmacokinetic models. Table S2. Muscle AIC values from fitting 0-45 min dynamic [18F]FMISO PET readings at baseline and week 2 to four different pharmacokinetic models. Figure S1. Example of the ROI from which measurements were taken. Figure S2. Examples of TAC in tumour, blood and muscle at baseline over 0-4 h. Figure S3. Example of 0-45 min tumour TACs fitted to four different pharmacokinetic models. Figure S4. Example of 0-45 min muscle TACs fitted to four different pharmacokinetic models. Figure S5. The artwork shows examples of scatter & random events originating from the activity inside the bladder as a potential cause of spill-in counts inside the tumour. Figure S6. An example from the enema group showing transaxial PET/CT images at 2 and 4 h. AIC=akaike information criteria; min= minute; [18F]FMISO=[18F]fluoromisonidazole; PET=positron emission tomography; CRT=chemoradiotherapy; ROI=region of interest; TAC=time activity curve; h=hour; CT=computed tomography. (PDF 445 kb) [file 13550_2017_324_MOESM1_ESM.pdf]

Additional supplementary file: [^{18}F]Fluoromisonidazole PET in rectal cancer

Patient	Baseline				After 8-10 fractions of CRT			
**Number**	**1 TCM**	**2 TCM**	**3 TCM**	**Casciari**	**1 TCM**	**2 TCM**	**3 TCM**	**Casciari**
P1	26	-20	-19	-71	6	-34	-29	-39
P2	86	44	47	-21	70	61	66	0
P3	50	73	17	-38	50	27	31	-52
P4	36	-23	18	-70	40	39	44	5
P5	63	42	39	-19	58	56	61	-17
P6	50	5	9	17	-	-	-	-
P7	84	65	72	-36	126	118	124	74
P8	27	8	6	-8	26	3	9	-5
P9	8	-10	-6	1	8	1	7	-4
P10	-	-	-	-	-	-	-	-
P11	65	30	30	-53	82	Failed	Failed	80

Table S1: Tumour AIC values from fitting 0-45 min dynamic [^{18}F]FMISO PET readings at baseline and after 8-10 fractions of CRT to four different pharmacokinetic models. AIC is used to provide assessment of how well the data fits to the model. Best fit is indicated by the lowest number (including negative). Table shows that 17 out of 19 times the Casciari model fitted the tumour data better than 1-, 2-, 3-TCM. AIC=akaike information criteria; min=minute; [^{18}F]FMISO=[^{18}F]Fluoromisonidazole; PET=positron emission tomography; CRT=chemoradiotherapy; TCM=tissue compartmental model.

Patient	Baseline				After 8-10 fractions of CRT			
**Number**	**1 TCM**	**2 TCM**	**3 TCM**	**Casciari**	**1 TCM**	**2 TCM**	**3 TCM**	**Casciari**
P1	27	31	37	-137	-20	-41	-36	-106
P2	-27	-69	-65	-157	-53	-46	-39	-132
P3	-5	1	8	-195	19	-9	-5	-159
P4	28	-46	-43	-152	14	-17	-12	-78
P5	38	42	49	-117	10	15	21	-17
P6	31	35	41	-73	-	-	-	-
P7	-23	-17	-12	-101	23	28	36	-64
P8	-30	-24	-7	-71	34	41	47	-125
P9	12	15	27	-122	0	-31	-16	-69
P10	-	-	-	-	-	-	-	-
P11	-2	-1	4	-114	-5	-40	-39	-18

Table S2: Muscle AIC values from fitting 0-45 min dynamic [^{18}F]FMISO PET readings at baseline and after 8-10 fractions of CRT to four different pharmacokinetic models. AIC is used to provide assessment of how well the data fits to the model. Best fit is indicated by the lowest number (including negative). Table shows that 18 out of 19 times Casciari model fitted the muscle data better than 1-, 2-, 3-TCM. AIC=akaike information criteria; min=minute; [^{18}F]FMISO=[^{18}F]Fluoromisonidazole; PET=positron emission tomography; CRT=chemoradiotherapy; TCM=tissue compartmental model.

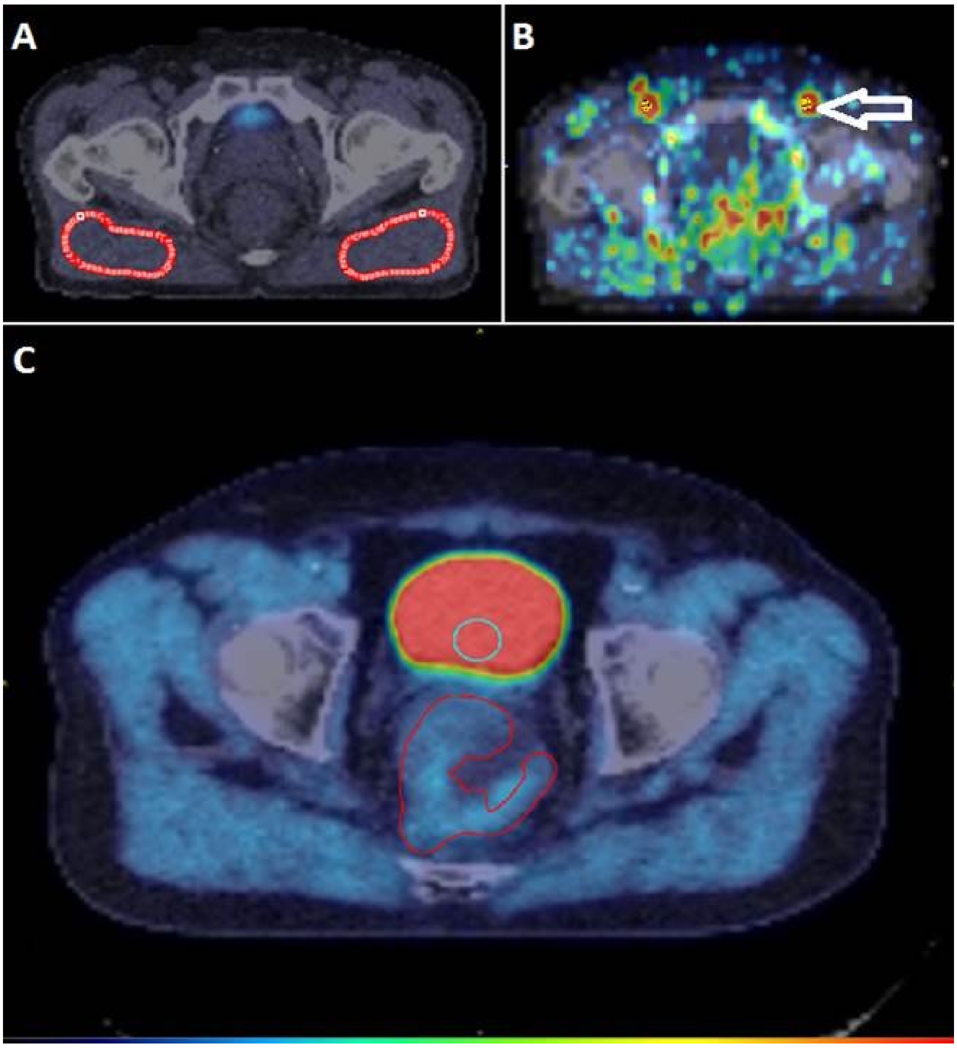

Figure S1: Example of the ROI from which measurements were taken. (A) The muscle ROI in red outline was drawn on PET-CT transaxial slices at 4 h. (B) A small circular ROI (in yellow indicated by the white arrow) was drawn on an early dynamic PET frame to mark the femoral artery. The combined activity concentration from both arteries was used to define the blood curve. (C) The bladder ROI was outlined on the PET scan is shown in blue. The tumour ROI in red outline was drawn on MRI and transferred to PET-CT trans-axial slices at 4 h. ROI=regions of interest; PET=positron emission tomography; CT=computed tomography; hr=hour; MRI=magnetic resonance imaging.

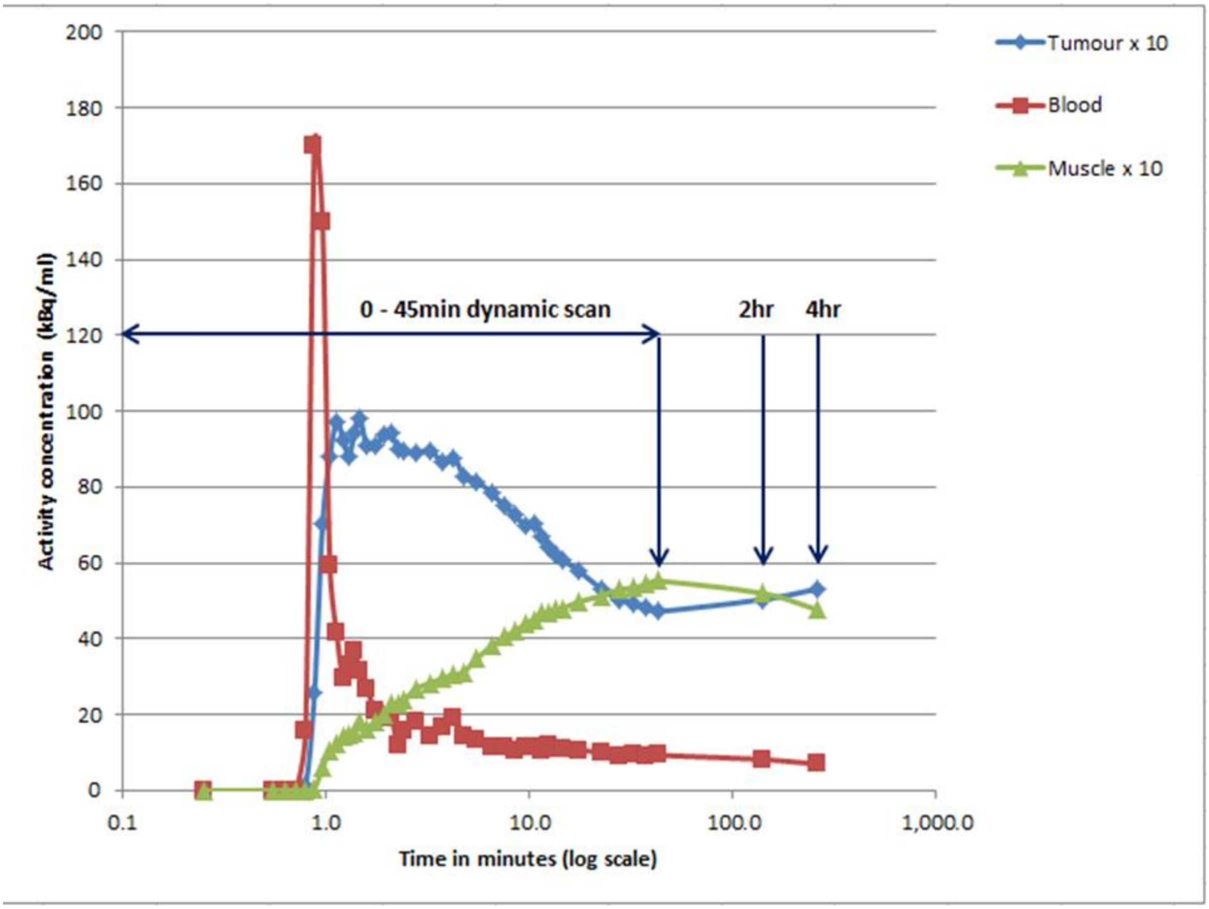

Figure S2: Examples of the [^{18}F]FMISO TAC in tumour, blood and muscle over 0-4 h at baseline. Tumour and muscle TACs are image derived, blood TACs combine data from image derived and blood sample activity. [^{18}F]FMISO=[^{18}F]fluoromisonidazole; TAC=time activity curve; h or hr =hour; CRT=chemoradiotherapy; kBq=kilo Becquerel; ml=millilitres; min=minute.

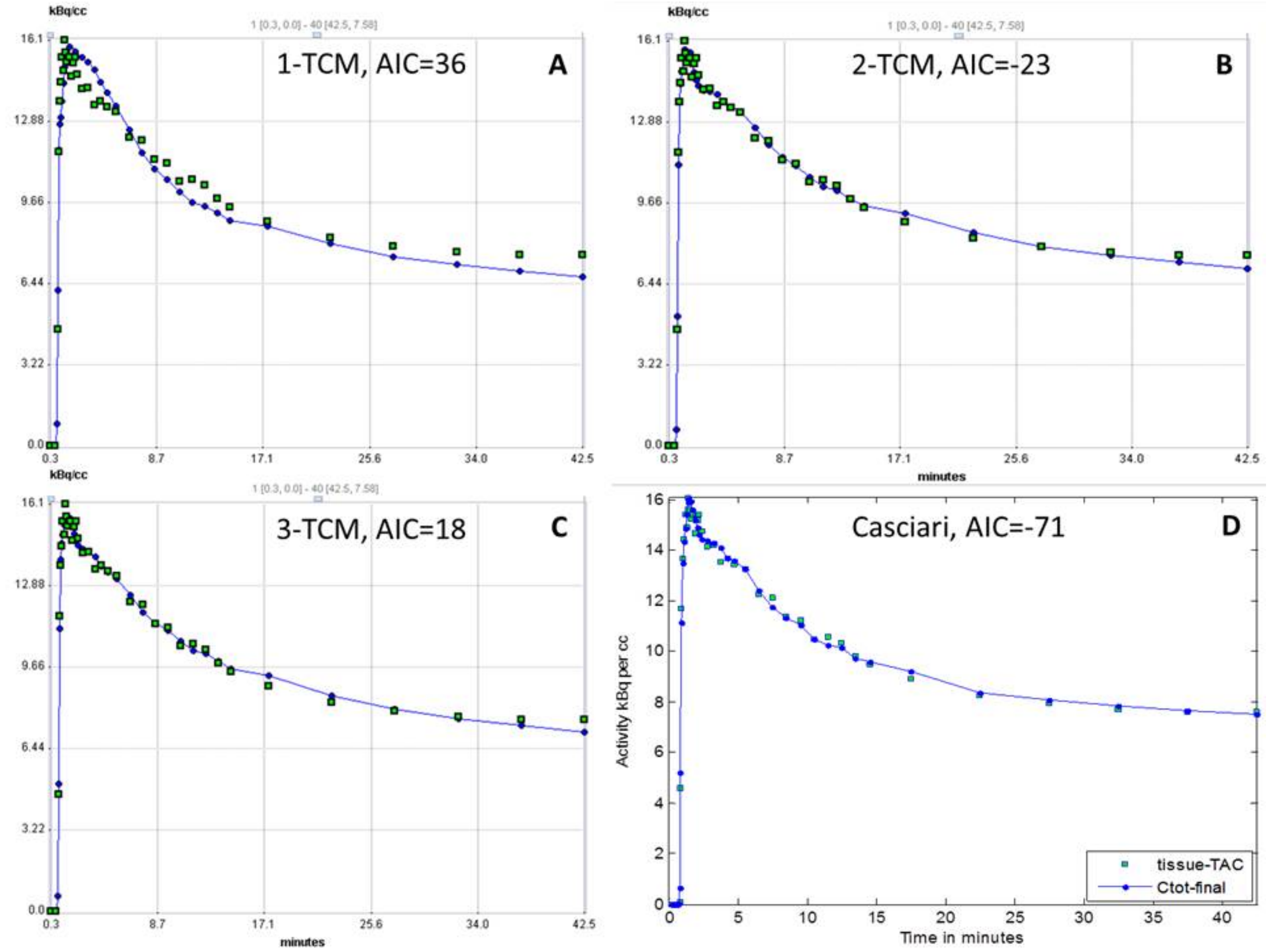

Figure S3: Example of 0-45 min tumour TACs fitted to four pharmacokinetic models: (A) 1-TCM; (B) 2-TCM; (C) 3-TCM and (D) Casciari model. TCM=tissue compartmental model; TAC= time activity curves; AIC=akaike information criteria; min=minute.

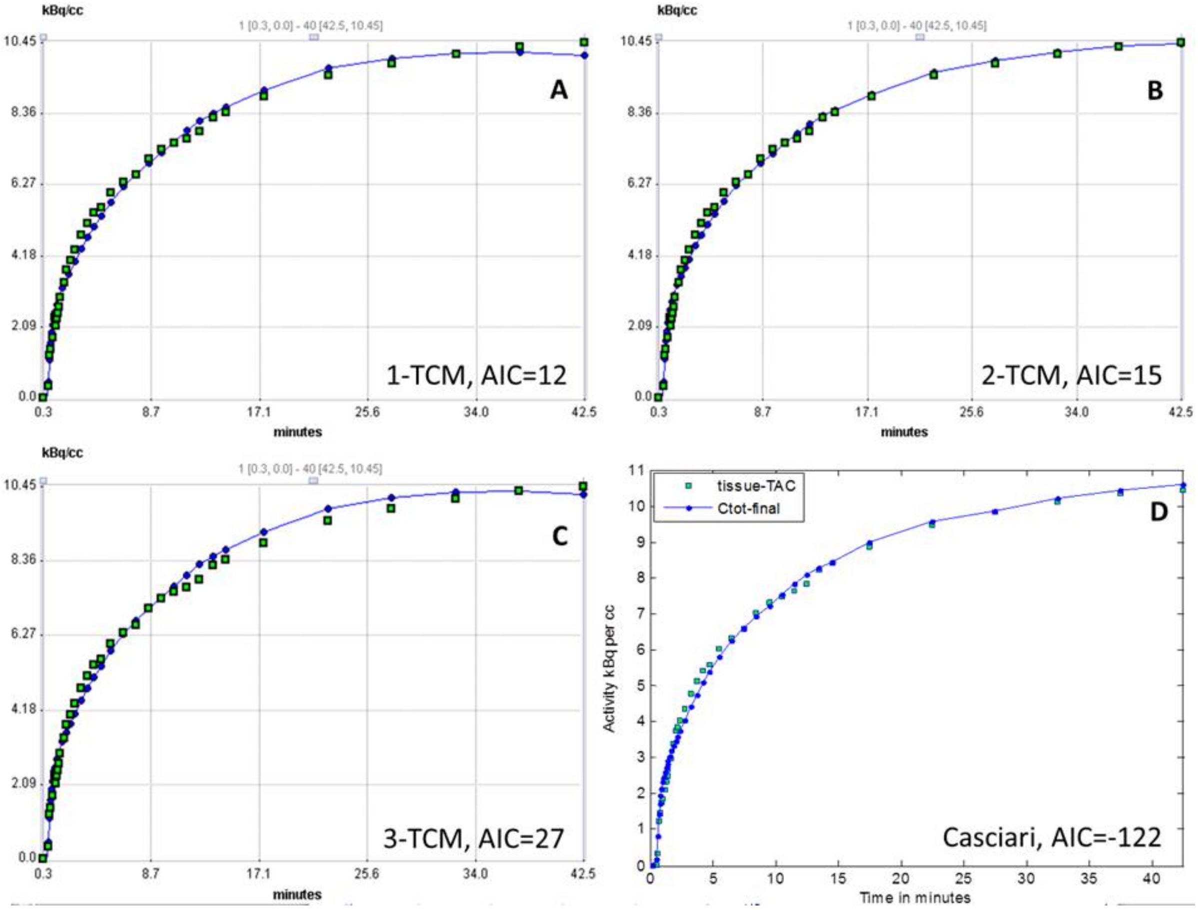

Figure S4: Example of 0-45 min muscle TACs fitted to four pharmacokinetic models: (A) 1-TCM; (B) 2-TCM; (C) 3-TCM and (D) Casciari model. TCM=tissue compartmental model; TAC= time activity curves; AIC=akaike information criteria; min=minute.

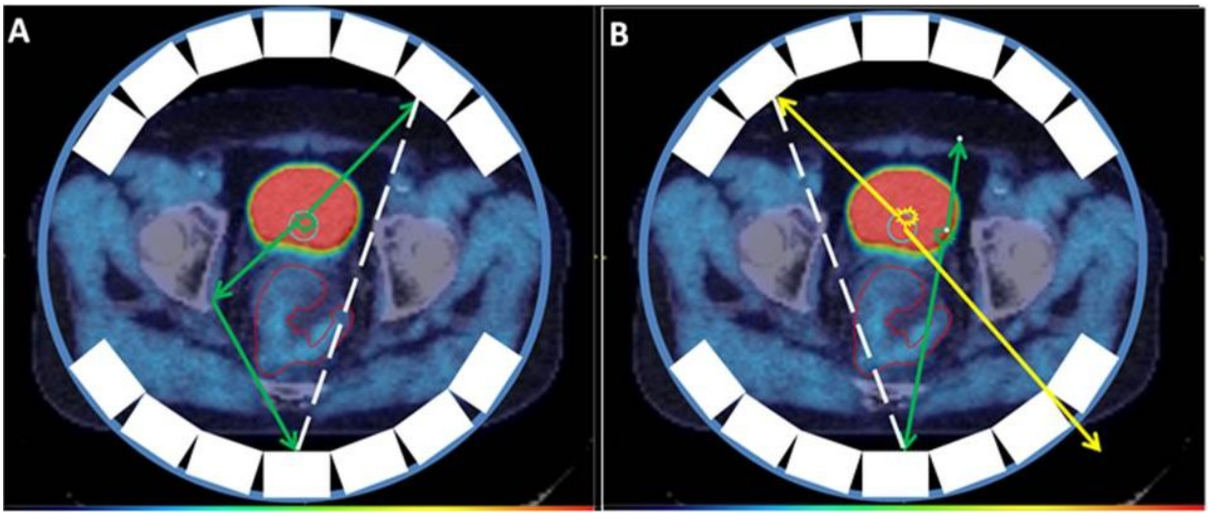

Figure S5: The artwork shows examples of scatter & random events originating from the activity inside the bladder as a potential cause of spill-in counts inside the tumour. The blue circular ring illustrates the PET scanner field of view, the white rectangular blocks represents the photon detectors, green and yellow arrows describe the path of the photons from two different annihilation events, red boundary represents tumour ROI and white dashed line shows the LOR. (A)An annihilation event occurs in the bladder and the path of a scattered photon is shown using green arrows. The two photons are detected between a pair of opposite detectors in 511 keV energy coincidence window and their LOR (white dashed line) passes through tumour falsely contributing to the image as a true event. (B)Two annihilation events occur in the bladder and one of the photons in each annihilation gets lost (or undetected) and the other two photons (one from each event) are detected between a pair of opposite detectors in 511 keV energy coincidence window and their LOR (white dashed line) passes through tumour (red boundary) falsely contributing to the image as a true event. PET=positron emission tomography; LOR=line of response; keV=kilo electron volt; ROI=region of interest.

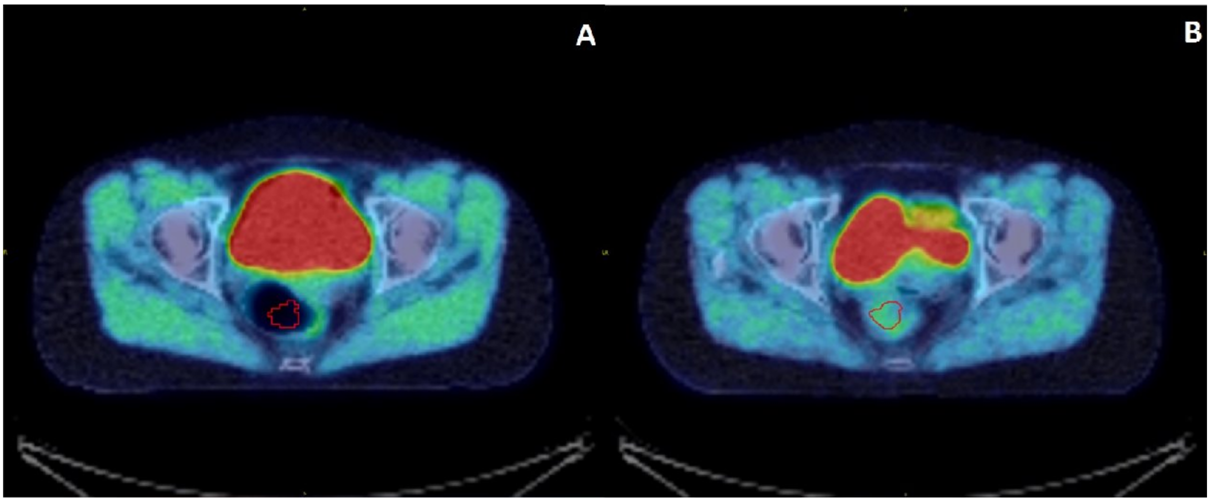

Figure S6: An example from the enema group showing transaxial PET-CT images at: (A) 2 h and (B) 4 h. When the tumour ROI drawn at 4 h was transferred to the 2 h image organ motion was seen showing that the tumour ROI was misplaced due to air in the subject’s rectum. This example shows that care is required in the ROI transfer procedure and that the scan images at each time-point may benefit from careful delineation of tumour region by a clinician. It is also clear that the patient emptying their bladder after the 2 h scan and clearance of air from the bowel made it easier to visually identify the tumour on the 4 h scan. The clearance of tracer from the surrounding muscle can be clearly seen at 4 h compared to 2 h. PET=positron emission tomography; CT=computed tomography; h=hour; ROI=region of interest.
